# *Echinococcus granulosus* genomics: a new dawn for improved diagnosis, treatment, and control of echinococcosis[Fn FN1]

**DOI:** 10.1051/parasite/2014066

**Published:** 2014-12-17

**Authors:** Wenbao Zhang, Shengyue Wang, Donald P. McManus

**Affiliations:** 1 State Key Laboratory Incubation Base of Xinjiang Major Diseases Research, Clinical Medical Research Institute, The First Affiliated Hospital of Xinjiang Medical University Urumqi PR China; 2 Shanghai–Ministry of Science and Technology Key Laboratory of Health and Disease Genomics, Chinese National Human Genome Center at Shanghai Shanghai PR China; 3 Molecular Parasitology Laboratory, QIMR Berghofer Medical Research Institute Brisbane Queensland Australia

**Keywords:** *Echinococcus granulosus*, Cystic echinococcosis, Cestodes, Genomics, Drug design, Zoonosis control

## Abstract

Cystic echinococcosis (CE) is a cosmopolitan disease caused by the dog tapeworm *Echinococcus granulosus*. The disease is difficult to diagnose, treat, and control and is responsible for considerable human morbidity and mortality globally. There is an urgent need for new diagnostic tests and new drugs for treatment of CE and the development of a vaccine against adult worms of *E. granulosus* in dogs. We recently presented a draft genomic sequence for the worm comprising 151.6 Mb encoding 11,325 proteins. We undertook an extensive comparative analysis of the *E. granulosus* transcriptome using representative life stages (protoscoleces, cyst germinal cells and membranes, adult worms, and oncospheres) to explore different aspects of tapeworm biology and parasitism. The genome and transcriptome of *E. granulosus* provide a unique platform for post-genomic research and to facilitate the development of new, effective treatments and interventions for echinococcosis control.

## Abstract (fr)

L’échinococcose kystique (EK) est une maladie cosmopolite due à l’infection par le ténia du chien *Echinococcus granulosus*. La maladie est difficile à diagnostiquer, à traiter et à contrôler; elle est responsable au plan mondial d’une considérable morbidité et mortalité. Il est urgent de découvrir de nouveaux tests diagnostiques et de nouveaux médicaments pour traiter l’EK, ainsi qu’un vaccin contre le développement des vers adultes d’*E. granulosus* chez le chien. Nous avons récemment publié la séquence génomique de ce parasite, qui comprend 151.6 Mb codant 11,325 protéines. Nous avons aussi analysé le transcriptome et comparé l’expression génique dans les principaux stades parasitaires (protoscolex, cellules germinatives et membranes du kyste, vers adultes et oncosphères) pour explorer différents aspects de la biologie et du parasitisme de ce cestode. La connaissance du génome et du transcriptome d’*E. granulosus* représente une base sans précédent pour le développement de recherches post-génomiques et leur application à la découverte de traitements novateurs et d’outils de prévention.

## Introduction

Cystic echinococcosis (CE), caused by the dog tapeworm *Echinococcus granulosus*, is responsible for considerable human morbidity and mortality globally [8, 9]. This parasitic disease is listed as one of the neglected tropical diseases in WHO’s “Accelerating work to overcome the global impact of neglected tropical diseases – a roadmap for implementation” [15]. This cosmopolitan disease is difficult to diagnose, treat, and control [3, 5, 6, 17, 20]. There is an urgent need for effective new drugs for human CE treatment and a vaccine effective against adult worm infections in dogs [3, 6]. A better understanding of the *E. granulosus* genome and gene expression/regulation is essential for developing new treatments and interventions.

In 2013, two complementary landmark and revolutionary papers describing the *E. granulosus* and *E. multilocularis* genomes were published. Tsai et al. [13] described a high-quality genome for *E. multilocularis*, together with draft genomes of three other tapeworm species including *E. granulosus*. Zheng et al. [21] reported the sequence and analysis of the *E. granulosus* genome and transcriptome. The two studies provide a rich source of information that gives new insights into the biology, differentiation, development, evolution, mechanisms of pathogenesis, and host interaction of *E. multilocularis* and *E. granulosus*. Further, these comprehensive data sets can facilitate the development of urgently needed new echinococcosis public health intervention tools given the inefficiencies of currently available drugs, the lack of appropriate diagnostic procedures, and the current difficulties in treatment and control.

## History of the project

Soon after the completion of the draft genomic sequence for *Schistosoma japonicum* in 2009 [22], the same collaborative groups, including the China Human Genome Center at Shanghai (CHCG) and the Queensland Institute of Medical Research (QIMR), Brisbane, Australia, together with Xinjiang Veterinary Research Institute of Xinjiang Academy of Animal Science and The First Affiliated Hospital of Xinjiang Medical University, Urumqi, Xinjiang, China, decided to use the same platform and strategy to sequence the transcriptome and genome of *E. granulosus.* The two Xinjiang institutes immediately prepared all the parasite materials required for the project. Initially, we used a 454 sequencer to obtain cDNA sequences for four stages of *E. granulosus*, namely the protoscolex, adult worm, oncosphere, and cyst membrane. Then, we prepared genomic DNA from a single (clone) large cyst from a sheep liver and used the DNA to decipher the complete genomic sequence. Totally, we obtained 2.8 Gb of 454 GS FLX shotgun sequences and 20.8 Gb of Solexa paired-end or mate-paired sequences, which assembled 967 scaffolds.

The draft genomic sequence for *E. granulosus* is 151.6 Mb in size and we predicted a total of 11,325 protein-coding genes, spanning only one tenth of the complete genome. Of these genes, 4,569 encoded proteins that were annotated by gene ontology (GO) terms, which allowed us to undertake additional gene analysis.

## *E. granulosus* has lost many genes associated with the synthesis of proteins and nucleic acids

Comparisons of the *E. granulosus* genomic sequence with those of six helminth taxa, comprising four parasite species (*Schistosoma japonicum*, *S. mansoni*, *Brugia malayi*, *Trichinella spiralis*) and two free-living nematodes (*Caenorhabditis elegans*, *Pristionchus pacificus*), indicated that the cestode has lost genes responsible for the synthesis of most amino acids and all purines and pyrimidines, molecules critical for basic metabolic processes. Similarly, *E. multilocularis* has lost the ability of synthesis of most amino acids [13]. On the other hand, *E. granulosus* has a number of genes encoding proteases (Supplementary Table 19 in Ref. [21]), that can digest host proteins, and a range of solute carrier family proteins for transporting amino acids from the mammalian host, indicating a close host-parasite relationship. We compared the protein domain profiles of *E. granulosus* with those of six other worms and two mammalian hosts (human and dog) to identify genes associated with parasitism. We identified 6,428 Pfam domains with 3,405 present in *E. granulosus*, of which about 20 are *E. granulosus-*specific.

These comparisons also showed that *E. granulosus* has acquired a spectrum of genes, including the EgAgB family, whose products are secreted by the parasite to interact and redirect host immune responses, and are a good antigenic resource for diagnosis.

## New intervention targets

BLAST analysis indicated 3,903 genes present in *E. granulosus* without gene homologues or orthologous groups in other taxa, suggesting that these are probably *Echinococcus-*specific. It is likely that these genes are the key genes responsible for the unique features and biological characteristics of *E. granulosus.* The products of these genes may also be of value as new candidates for diagnosis and drug and vaccine targets for *E. granulosus* and CE. The comparison of the genomes of the two *Echinococcus* parasites showed a high similarity in sequences, indicating that the two parasites may share the same molecular targets for vaccine and drug development (data not shown).

## Vaccine candidates

There were 340 genes highly up-regulated among the 3,811 genes expressed in oncospheres compared with those in adults and the cyst of *E. granulosus*. The up-regulated genes accounted for 55.9–61.8% of the transcript reads (Supplementary Table 28 in Ref. [21]) in oncospheres, of which genes encoding secreted proteins were predominant. We found 74 genes encoding secreted proteins, and among them was the eg95 family comprising seven genes and having 4% of the oncospheral transcript reads. Vaccination of sheep with eg95 has been shown to generate more than 95% protective efficacy following experimental egg challenge infection. Other proteins, such as protease inhibitors and tetraspanins, that were highly and specifically expressed in oncospheres, likely represent additional vaccine candidates for echinococcosis (Supplementary Table 47 in Ref. [21]).

It is probable that a successful echinococcosis control program will require a vaccine effective against the adult worm infection in dogs [3]. The protoscolex is the infective stage for dogs and attacking the mechanism of its attachment to the dog intestine is a likely target for vaccine development. Neurotransmitters, neuropeptides, tegument, and secreted proteins, including tetraspanin 1 (EG_10196) and a protease inhibitor, were specifically expressed in adult worms, which may play a role in adaption or evasion from host attack and these are likely vaccine candidates for further study.

It has been shown that dog bile acids play an important role in the differentiation of the protoscolex to the adult worm. It is probable that the proteins associated with bile acid transport and nuclear hormone receptors for bile acid signaling (EG_00119, EG_00780, EG_04405, and EG_08428) represent putative vaccine candidates against adult worm development in the dog host. Further, we identified 92 genes encoding sensory system elements (Supplementary Table 40 in Ref. [21]) as additional vaccine targets. These included homologs associated with taste and smell, such as olfactory receptor, G protein, and adenylate cyclase type 3.

## “Druggable” targets

The current treatment of CE involves surgery and the use of benzimidazole drugs (5), but the results are far from satisfactory, and new drug compounds are urgently needed [1, 11, 14].

To identify new potential drug targets, we surveyed common targets of existing pharmaceuticals including G-protein coupled receptors (GPCRs), serine/threonine and tyrosine protein kinases, zinc metallopeptidases, serine proteases, and nuclear hormones. We listed the possible targets for echinococcosis treatment based on the transcription of these genes in the cystic stage (Supplementary Tables 44–45 in Ref. [21]). Other classes, such as ion channels, lipid metabolism components, ligand- and voltage-gated ion channels and neuropeptides, may also represent druggable targets (Supplementary Tables 46 in Ref. [21]).

*E. granulosus* and the other parasitic worms encode a special orthologous group of prenylcysteine oxidases (EG_06057), which may catalyze the final step in the degradation of prenylated proteins. Prenyltransferase is a key enzyme in the biosynthesis of prenylated proteins and, along with prenylcysteine oxidase, may also represent a further novel drug target.

Glutamate receptors are major therapeutic targets of anthelmintic therapy and the avermectins (including ivermectin), which inactivate the alpha-subunit of glutamate-gated chloride channels with high affinity, have shown activity against a broad range of adult helminth worms [4, 10, 12, 16, 18]. We identified in the *E. granulosus* genome a range of transient receptors including LDL receptors [2], which have also shown potential as targets for drug discovery [7].

## Diagnostic targets

Although diagnostic imaging techniques have been widely used in detecting echinococcocal cysts, improved immunodiagnosis is needed, especially for early CE diagnosis and post-management (i.e. follow-up of the patients after interventional or drug treatment). However, this area is still problematic due mostly to the lack of specific antigens. As we have indicated earlier, EgAgB (antigen B) is an *E. granulosus*-specific family of seven genes, whose products are secreted and have been extensively studied as diagnostic molecules for CE. We found 809 proteins that were extracellular or secreted (Supplementary Table 41 in Ref. [21]). These proteins likely serve as messengers for cross-talk between *E. granulosus* and its mammalian hosts, playing key roles in regulating host immune responses. Genes for a number of secreted products from *E. granulosus* are expressed highly in the oncosphere and hydatid cyst (Supplementary Table 28 in Ref. [9]). Some of these proteins, including antigen B [19], have shown some promise in CE serodiagnosis. We have listed candidates as new serodiagnostic targets using the following criteria: extracellular or containing extracellular domain proteins secreted in the oncosphere/cyst stage with high transcript reads, high immunogenicity, and minimal homology to their mammalian counterparts (Supplementary Table 48 in Ref. [21]).

## Post-genomics – What comes next?

The aim of parasite-omics is to understand parasite biology and the functional basis of host-parasite interactions but, most importantly, to improve diagnosis and to discover new drug and vaccine targets against the diseases they cause.

The publication of the genome and transcriptome of *E. granulosus* provides the platform, not only for deeper understanding of the molecular biology and physiology of this parasite and for illuminating mechanisms of pathogenesis in echinococcosis, but also for developing the new public health interventions against echinococcosis that are urgently required, given the inefficiencies of currently available drugs, the lack of appropriate diagnostic procedures, and the current difficulties in treatment and control.

Further research directions include:

- *E. granulosus* has a complex life cycle, whose developmental stages alternate between intermediate (wild or domesticated ungulates, such as sheep) and definitive hosts (dogs and foxes) (humans are accidental hosts). Up- or down-regulation of gene expression likely underpins the phenotype changes associated with the different life cycle stages. In-depth transcriptomic analysis is critical for searching for the key genes correlating with these changes and for identification of their specific function.
- *E. granulosus* and *E. multilocularis* have differing biological characteristics, clinical features, pathologies, and host preferences. A comprehensive comparison of the genomes and transcriptomes of the two species is central to our understanding of these differences.
- Evolutionary genomics combined with stage-specific characterization may play a role in predicting the possible function of novel genes in *E. granulosus* and *E. multilocularis*. Besides reconstructing phylogenetic relationships, evolutionary frameworks have been applied to improve functional annotation of genes and gene products, as well as to study gene/protein family evolution for many diverse taxa.
- The post-transcriptional suppression of genes through RNA interference (RNAi) techniques may promote gene function identification in *E. granulosus*.
- The genome/transcriptome information should be used in the identification of some important molecules for serodiagnosis and drug and vaccine development for *E. granulosus* as a priority to improve the diagnosis, treatment, and control of CE.

In conclusion, the availability of the *E. granulosus* and *E. multilocularis* genome and transcriptome data provides the platform and insight for many areas of research, not only for understanding the biology and evolution of *Echinococcus*, but also by representing an invaluable resource to the research community to develop much needed new control interventions for the treatment and elimination of echinococcosis globally.
